# Retrospective, single-centre analysis comparing clinical outcomes of reverse total shoulder replacement for fracture, degenerative changes and revision procedure

**DOI:** 10.1186/s12891-025-09329-w

**Published:** 2025-11-14

**Authors:** Patricia Bergert, Ralf Henkelmann, Pierre Hepp, Jan Theopold

**Affiliations:** Department of Orthopedics, Trauma and Plastic Surgery, Division of Arthroscopic and special Joint Surgery/Sports Injuries, University of Leipzig, Liebigstrasse 20, Leipzig, 04103 Germany

**Keywords:** Reverse total shoulder arthroplasty, Indications, Outcomes, Questionnaire

## Abstract

**Background:**

Reverse total shoulder arthroplasty (RTSA) is a widely used treatment for both traumatic and degenerative glenohumeral joint diseases. Despite its popularity, data on outcomes sorted according to indications have revealed inconsistent results. Furthermore, the clinical outcome is rarely analysed in a differentiated manner using several scores.

This retrospective, single-center study aimed to investigate the impact of indications on the clinical outcomes of RTSA.

**Methods:**

The Constant Score, EQ-5D-5L, DASH Score were sent by post to 263 patients who received a RTSA between February 2011 and March 2022. The follow-up period was 12-139 months. Patients were categorized into primary-fracture, secondary-intervention, and degenerative-disease groups based on the indications for RTSA. We have calculated the corresponding average scores for each group.

**Results:**

Of the 263 patients included in the study, 136 completed the questionnaire. The mean follow-up duration was 48 months. All outcome measures were approximately normally distributed. The mean values were as follows. Constant Score: degenerative disease 58 ± 19, primary fracture 52 ± 18, secondary intervention 49 ± 18. DASH Score: degenerative disease 35 ± 20, primary fracture 40 ± 17, secondary intervention 42 ± 20. EQ-5D-5L Index: degenerative disease 0.72 ± 0.24, primary fracture 0.75 ± 0.20, secondary intervention 0.68 ± 0.39.

**Conclusions:**

In this study, clinical outcomes after RTSA varied according to the underlying indication, with the best results observed in patients with degenerative diseases and the lowest in patients undergoing secondary interventions. Further studies with larger, multi-centre cohorts are needed to confirm these findings and to strengthen the evidence base before clinical recommendations can be made.

**Trial registration:**

593/21ek.

## Background

In recent years, the number of available endoprosthetic treatments has significantly increased worldwide. Reverse total shoulder arthroplasty (RTSA) is largely responsible for this increase [1]. RTSA has been the most frequently performed endoprosthetic procedure in the past decade [2].

Initially, RTSA was mainly performed in the elderly population for rotator-cuff tear arhtropathy [1]; Today, RTSA is a proven treatment option for both degenerative and trauma-associated diseases [3], and is particularly advantageous in terms of biomechanics; therefore, its application is increasing [4].

Currently, the indications for this treatment include complex proximal humeral fractures [5], various types of arthritis with bone loss of the glenoid fossa, tumors [6], and revision implant surgeries in patients with primary-implant failure.

Several studies have reported the outcomes of RTSA. However, there are only a few studies that differentiate the clinical outcome according to indication [7, 8]. Moreover, the available data are inconsistent [7, 8].Therefore, this study aimed to assess whether outcomes are influenced by the indications for RTSA. For this purpose, we used three different patient reported outcome measures.

## Methods

The study was approved by the responsible ethics committee (593/21-ek) and was conducted in accordance with the Declaration of Helsinki and the guidelines of the International Conference on Harmonization (Good Clinical Practice).

All patients who underwent RTSA between 03/02/2011 and 03/03/2022 were included in this single-center follow-up investigation. The ICD-10 diagnostic codes (M19.01; M19.11; S42.29; 5–824.21.21; 5–825.12.12; 5–825.21.21) were used to identify patients who had undergone RTSA during the study period from the hospital’s patient record system. All procedures were performed by three surgeons with extensive experience in the field of shoulder surgery.

**Fig. 1:**
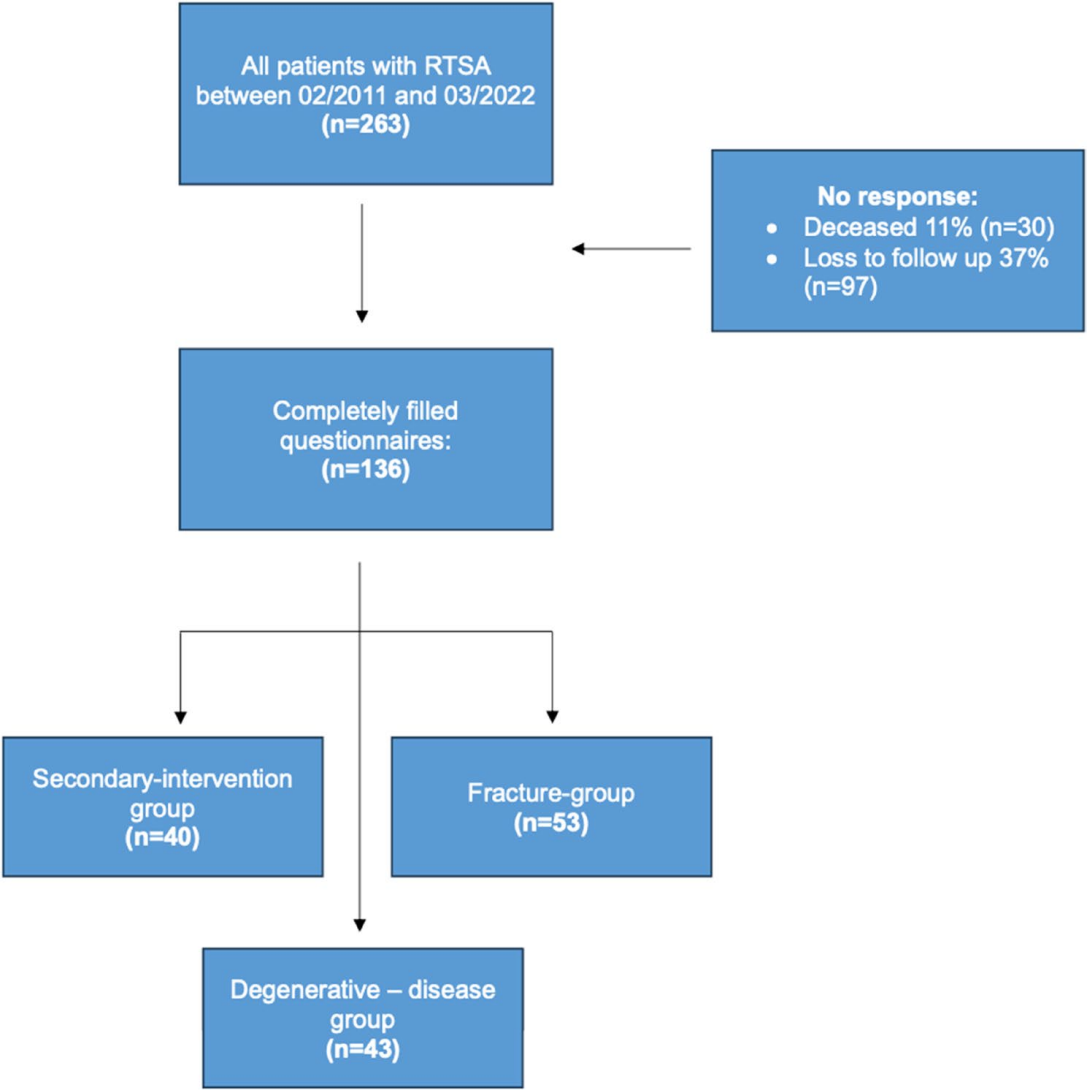
Flowchart for patient selection

Patients were divided into three groups based on the indications: primary fractures, secondary interventions, and degenerative diseases. The diagnosis of degenerative disease was made using magnetic resonance imaging. Fractures were identified on the basis of radiological imaging and assessed according to the Neer classification [9]. Secondary interventions included treatment for defects caused by trauma or infection and loosening of prostheses (Fig. 1).

We used the Constant-, the DASH- and the EQ-5D-5 L-test to record pain, range of motion and quality of life [10–12]. Because of the ongoing coronavirus pandemic and to minimize the risk of infection, we sent out the questionnaires by post and did not conduct an examination.

The questionnaires were sent between June 2022 and February 2023, however, at the earliest 12 months after implantation. If there was no response within 2 months, we contacted the patients again. If we received incomplete questionnaires, we contacted the patients and asked them to complete them.

Statistical analyses were performed using the IBM SPSS Statistics software, version 28 (SPSS Inc., Chicago, IL, USA). Due to the study’s explorative nature and the expected small sample size, we initially performed a descriptive statistical test. The data were approximately normally distributed; therefore, all outcome measures are presented as mean ± standard deviation.

The patient inclusion process is shown in Flowchart 1.

## Results

Between February 2011 and March 2022, a total of 263 patients underwent RTSA. Among these, 136 patients (52%; 89 female, 47 male) completed the questionnaire. Thirty patients (11%) had died by the time of the investigation, and 97 patients (37%) could not be contacted. Due to the long study period and the severity of the disease, it was not possible to contact more patients despite multiple attempts. Furthermore, a considerable number of patients had dementia and were unable to complete the questionnaires.

Some basic data on the indication groups are shown in Table 1.

**Table 1:** Overview and characterisation of the individual indication groups

	*Primary-fracture group* *n=53*	*Secondary-intervention group* *n=40*	*Degenerative-disease group* *n=43*
*mean age*	72.7 years (range, 40–86 years)	69.2 years (range, 48–85 years)	69.1 years (range, 52–83 years)
*gender (female/male)*	37/18	27/15	14/25
*follow-up time*	47 months	53 months	44 months
•*comorbidites* •*Hypertension* •*Diabetes mellitus* •*Cardiovascular disease* •*Pulmonary disease* •*Osteoporosis*	28 (53%) 10 (19%) 12 (23%) 6 (11%) 8 (15%)	22 (55%) 7 (18%) 11 (28%) 5 (13%) 9 (23%)	25 (58%) 6 (14%) 9 (21%) 7 (16%) 5 (12%)
*work-status* *(retired/employed)*	47/6	35/5	41/2

The mean age of the patients at the time of surgery was 71 ± 12 years. The overall mean follow-up period was 48 ± 32 months (range, 12–139 months). When stratified by indication, the mean follow-up was 50 ± 33 months in the fracture group, 46 ± 31 months in the secondary-intervention group, and 48 ± 32 months in the degenerative-disease group, with 19 patients having undergone RTSA more than 84 months earlier. The three indication groups were the fracture group (*n* = 53, 39%, mean age 72.7 ± 18 years), the secondary-intervention group (*n* = 40, 29%, mean age 69.2 ± 18 years), and the degenerative-disease group (*n* = 43, 32%, mean age 69.1 ± 19 years).

**Fig. 2:**
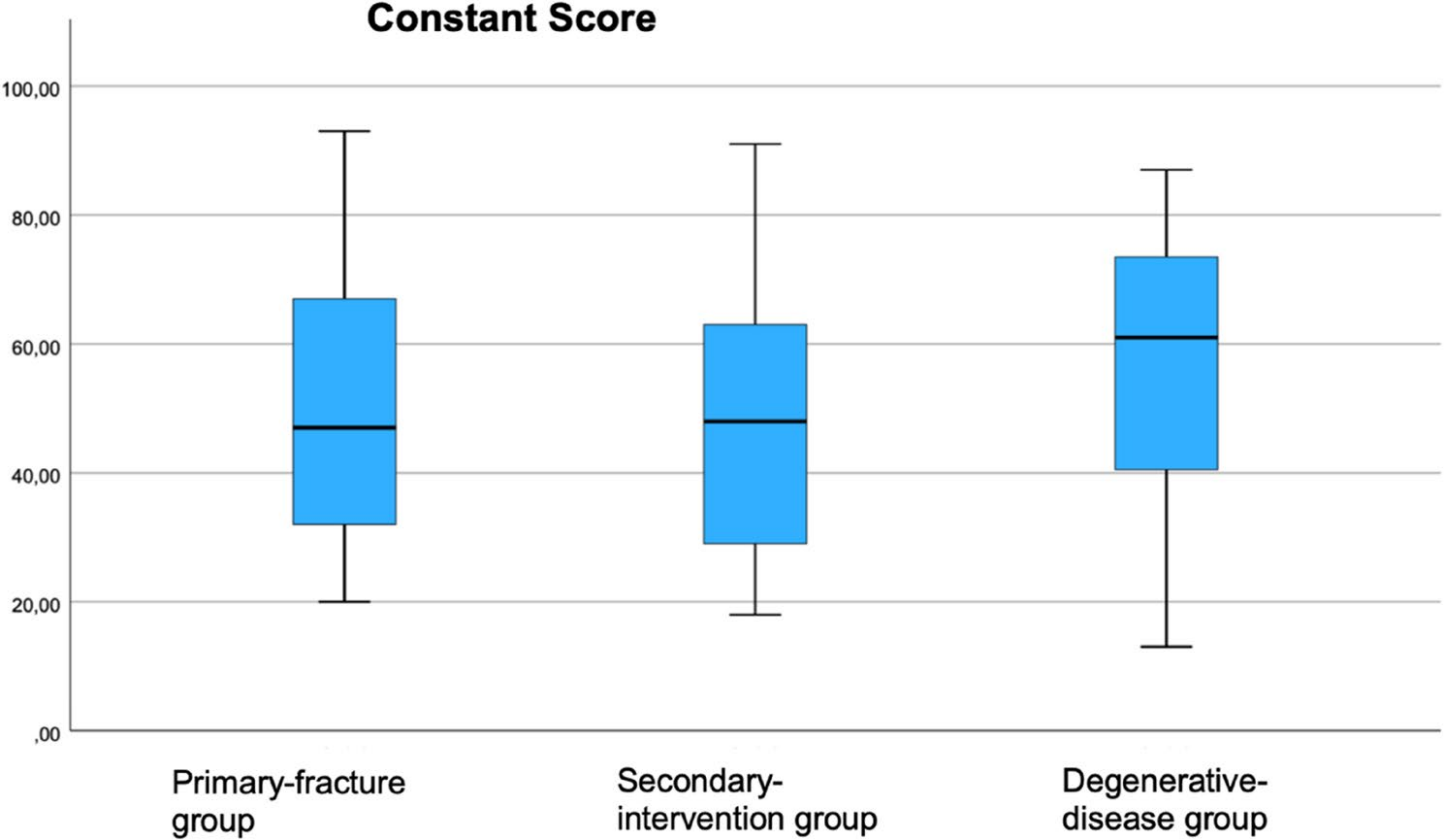
Clinical outcome: constant score of all three groups at least 12 months post-operatively

The mean Constant Scores were 58± 19 in the degenerative-disease group, 52 ± 18 in the primary fracture group, and 49 ± 18 in the secondary-intervention group. (Fig. 2)

**Fig. 3:**
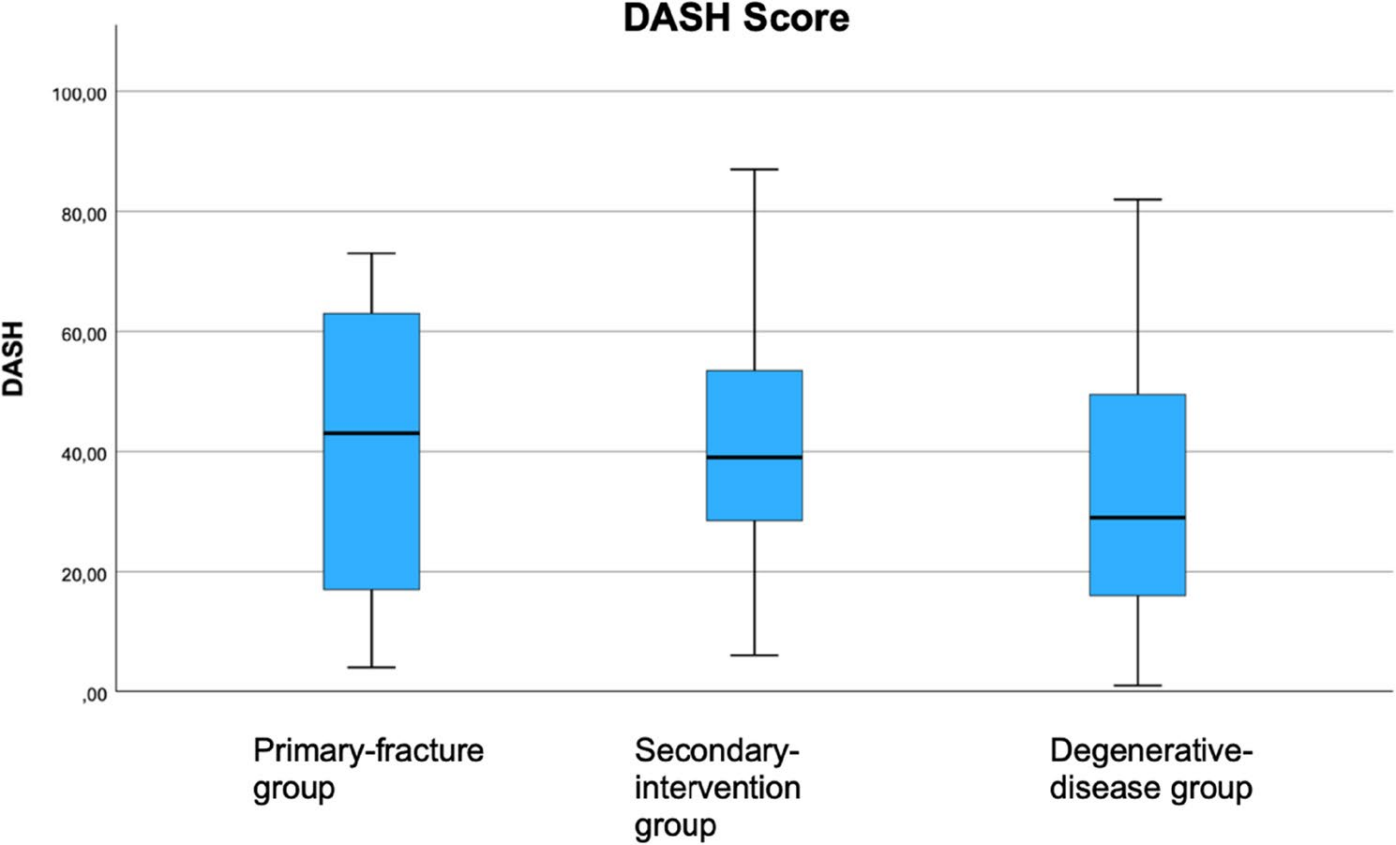
Clinical outcome: DASH Score of all three groups at least 12 months post-operatively

The mean DASH scores were 35 ± 20, 40 ± 17, and 42 ± 20 in the degenerative-disease, primary fracture, and secondary-intervention groups, respectively. (Fig. 3)

**Fig. 4:**
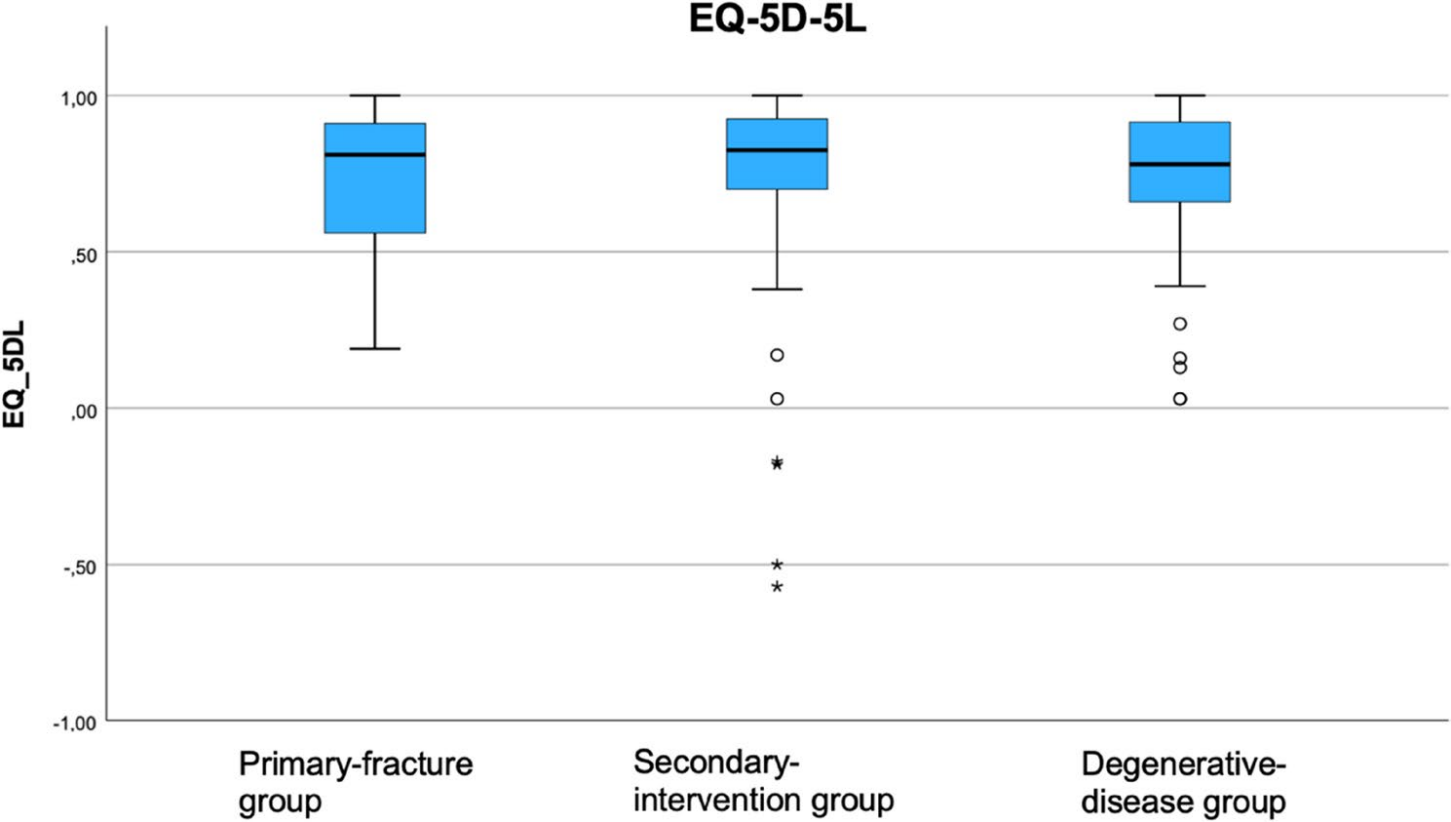
Clinical outcome: EQ-5D-5L of all three groups at least 12 months post-operatively

The mean EQ-5D-5 L Index values were 0.72 ± 0.24, 0.75 ± 0.20, and 0.68 ± 0.39 in the degenerative-disease, primary fracture, and secondary-intervention groups, respectively. (Fig. 4)

## Discussion

Our findings suggest that the indication may play a role in influencing clinical outcomes. More than half of the patients completed the follow-up questionnaire, resulting in a response rate above 50%, which is generally considered adequate for representativeness in postal follow-up studies [13, 14]. These results should, however, be interpreted with caution. While trends between the different indication groups were observed, the differences did not reach the threshold of clinical relevance. Given the limited sample size and the potential impact of confounding factors, definitive conclusions cannot be drawn. Nevertheless, the findings underline the need for larger, controlled studies to further investigate potential differences between indication groups.

In our study, the lowest outcomes were observed in the secondary intervention group, with a mean Constant score of 49 points, which is consistent with findings from Ortmaier et al. and Boileau et al. [15, 16]. By contrast, patients with degenerative disease and primary fractures achieved higher scores, though still lower than those reported by Boileau et al. [16]. Methodological differences may contribute, as Boehm et al. showed that self-assessed Constant scores tend to be around ten points lower than investigator-based scores [17]. Using standardized self-assessment, we were able to confirm the reproducibility of this approach.

There is a lack of studies analysing the clinical outcome of RTSA with scores other than the Constant score. Therefore, we additionally analysed the clinical outcome using the DASH score and the EQ-5D-5 L score and were able to show that indication-related differences are also visible in these instruments. Beyond indication alone, such PROMs may also be influenced by broader patient factors such as overall musculoskeletal status, preoperative strength and mobility, comorbidities, frailty, or falls risk. This underlines that measures like EQ-5D-5 L, as a global health-related quality of life score, or DASH, as an upper extremity functional score, capture not only shoulder-specific recovery but also the patient’s general health context. Our DASH results for primary fractures align with previous studies [18, 19], while in secondary interventions they were superior to those of Hartel et al. [20]. Compared with John et al., who reported 49 points at 24 months [21], our secondary intervention group achieved 58 points, though with longer follow-up. EQ-5D-5 L scores were also more favorable than in other reports [22].

Overall, our findings support previously reported trends: patients with degenerative disease often perceive greater postoperative benefit due to pre-existing limitations, fracture patients tend to judge recovery against a normal pre-injury state, and secondary intervention patients face the greatest challenges due to complex surgical histories. These baseline differences are crucial for interpreting outcomes after RTSA.

In their studies, Favard et al. and Bassens et al. reported a significant deterioration in Constant scores at eight and ten years post-operatively, respectively [22, 23]. In our cohort, 19 of 136 patients had undergone surgery eight years or more before inclusion, which may partly explain the lower scores in the primary-fracture and degenerative-disease groups compared with other studies. Although we did not adjust for time since surgery due to sample size constraints, this factor represents a potential confounder and should be considered when interpreting our results in comparison to studies with shorter follow-up periods.

This study has limitations, primarily its retrospective design, which led to case losses and prevented randomization or correction of incomplete data; to reduce bias, only fully completed questionnaires were analyzed. The limited sample size may have further reduced statistical power. Preoperative scores were not available, although the study’s focus was on differences between indications rather than surgical improvement. Finally, while self-assessment questionnaires are known to yield lower values than physician-based assessments, the large cohort and use of three validated instruments administered by mail represent a methodological strength.

## Conclusions

Clinical outcomes following RTSA varied according to the underlying indication, with patients with degenerative diseases achieving the highest scores and those undergoing secondary interventions the lowest. Further studies with larger, multi-centre cohorts are needed to confirm these findings and to strengthen the evidence before any clinical recommendations can be made.

## Data Availability

All data generated or analysed during this study are included in this published article.

## Abbreviation

RTSA: Reverse total shoulder arthroplasty
